# Granulomatous prostatitis mimicking prostate cancer in a patient with psoriatic arthritis: a case report

**DOI:** 10.2144/fsoa-2020-0031

**Published:** 2020-06-17

**Authors:** Luigi De Luca, Felice Crocetto, Biagio Barone, Massimiliano Creta, Salvatore Pesce, Achille Aveta, Maria Raffaela Campanino, Ciro Imbimbo, Nicola Longo

**Affiliations:** 1 Department of Neuroscience, Reproductive Sciences & Odontostomatology, University of Naples Federico II, Naples, Italy; 2 Department of Advanced Biomedical Sciences, University of Naples Federico II, Naples, Italy

**Keywords:** digital rectal examination, granulomatous prostatitis, multiparametric MRI, prostate imaging-reporting and data system, prostate specific antigen, psoriasis

## Abstract

Granulomatous prostatitis (GP) is an unusual and benign inflammatory condition of the prostate, where autoimmunity has been recognized as a key factor in the pathogenesis of GP in a subset of patients. Clinically, GP poses diagnostic challenges as it may strongly mimic prostate cancer from a clinical, biochemical and radiological point of view. The occurrence of GP in patients suffering from psoriasis, a systemic autoimmune disease, has never been investigated. We describe the case of GP in a patient with psoriatic arthritis presenting with an increased prostate specific antigen level, and evidence of a nodular lesion visualized by prostate multiparametric magnetic resonance imaging, which was highly suspicious for aggressive prostate cancer.

## Lay abstract

Granulomatous prostatitis is an uncommon inflammatory condition of the prostate that can mimic prostate cancer due to increased prostate specific antigen levels and suspect findings from both digital rectal exploration and prostate magnetic resonance imaging. This condition is considered an autoimmune disorder in many cases. We report the association between granulomatous prostatitis and psoriasis, another autoimmune disease.

Granulomatous prostatitis (GP) is an uncommon chronic inflammatory condition that accounts for approximately 3.3% of all benign inflammatory lesions of the prostate [1]. GP has been classified into nonspecific, specific, postsurgical and secondary to systemic granulomatous diseases [2–5]. Systemic diseases commonly reported to be associated with GP include sarcoidosis, rheumatoid arthritis, Wegener’s granulomatosis, polyarteritis nodosa and Churg–Strauss syndrome [2–4]. Nonspecific GP is considered an autoimmune disease characterized by a T-cell response against proteins in prostatic secretions [5]. Similarly, psoriasis is an autoimmune disorder that manifests as autoreactive T cells and is co-morbid with other autoimmune disorders [6]. There are no published evidences about the occurrence of PG in patients with psoriasis. We describe a case of GP mimicking prostate cancer (PCa) during clinical and radiological evaluations in a patient with psoriatic arthritis.

## Case presentation

**Figure 1.**
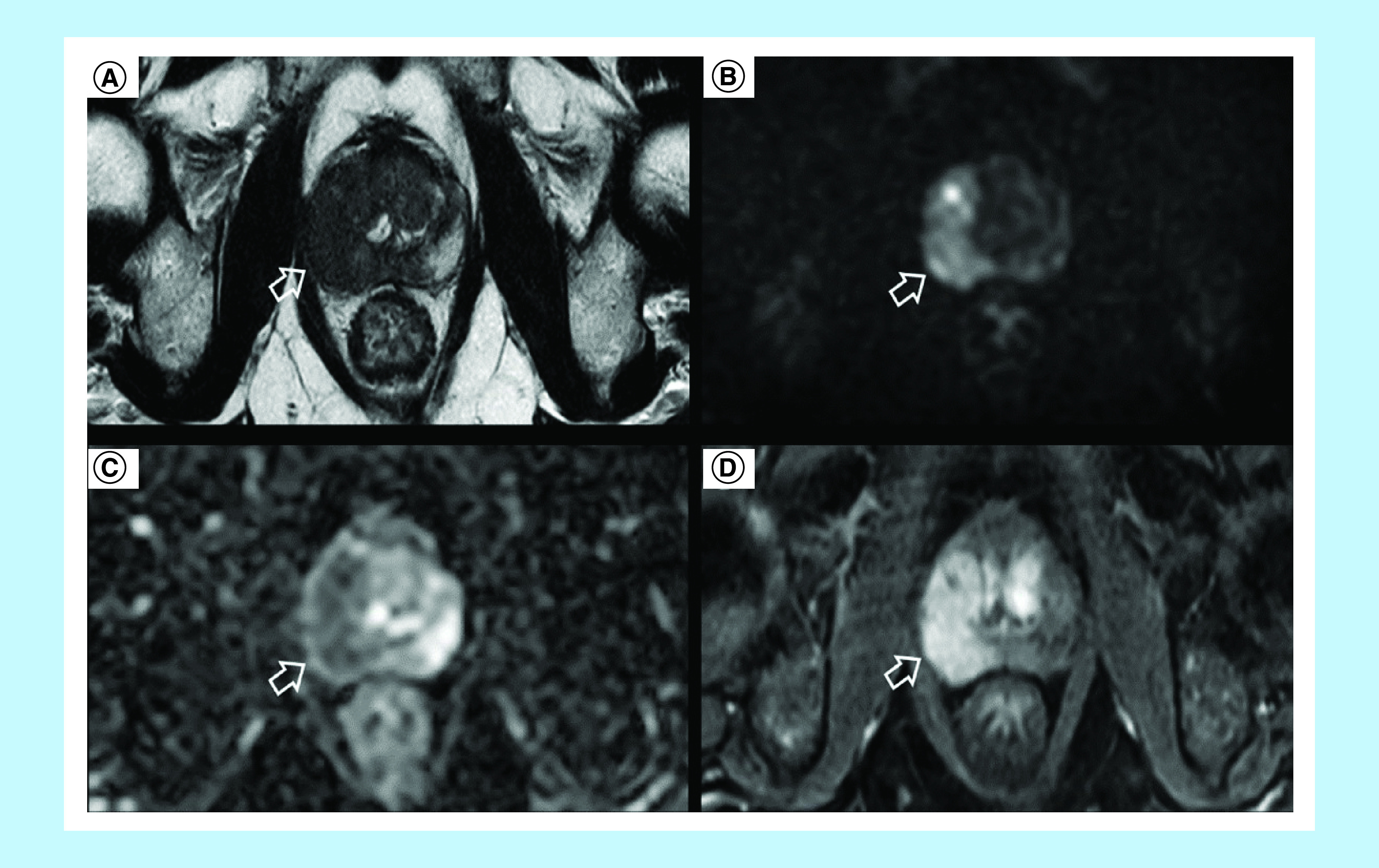
Multiparametric magnetic resonance imaging findings. **(A)** Axial T2WI scan demonstrating a large (4 cm) nodular lesion involving both peripheral and transition zone of the right lobe and extending to the peripheral zone of the mid basal left lobe with capsular irregularity suspicious for extracapsular extension. **(B)** Diffusion-weighted imaging demonstrating signal restriction. **(C)** Apparent diffusion coefficient map showing low apparent diffusion coefficient values. **(D)** Dynamic contrast enhanced images revealing diffuse postcontrast enhancement.

**Figure 2.**
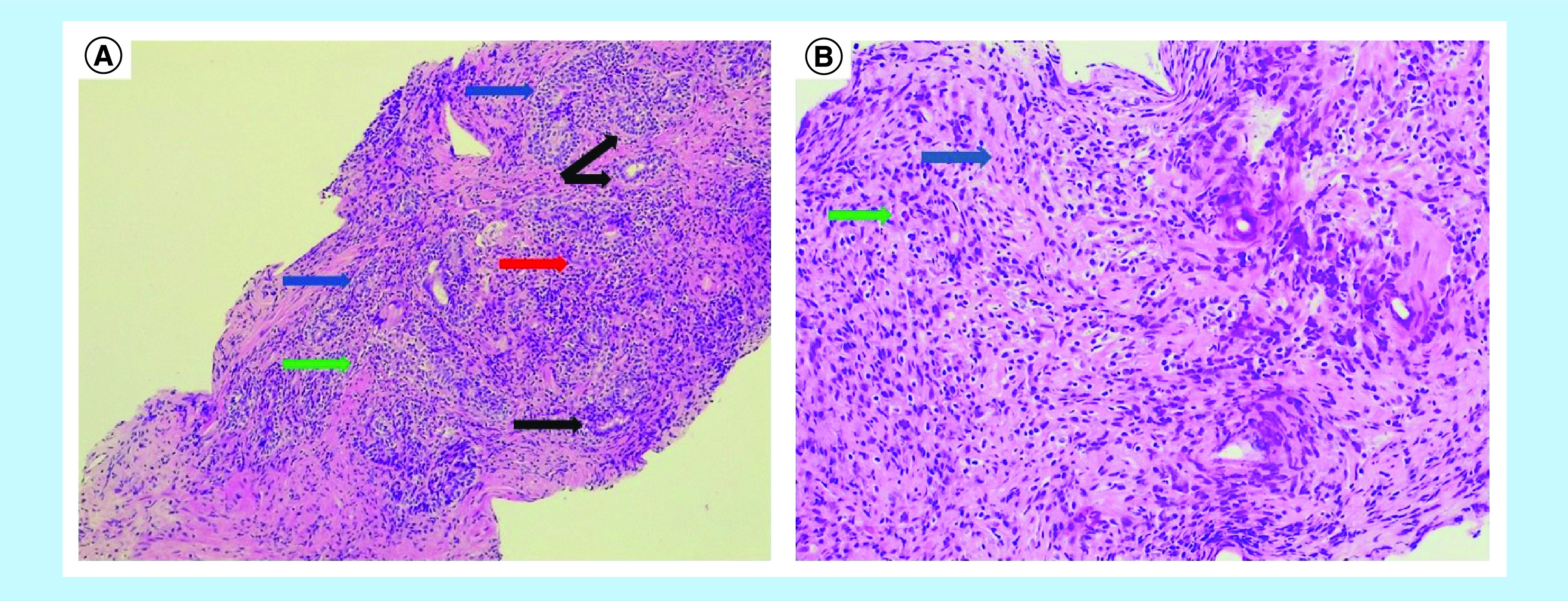
Histology of the biopsy specimen of the prostate. **(A)** Low (4×) and **(B)** high (40×) magnification. Hematoxylin and eosin images showing granulomatosus reaction characterized by atrophy and glandular prostatic damage (black arrows), aggregates of lymphocytes, plasmacells, histiocytes and epithelioid cells, typically around the damaged glands (blue arrows), multinucleated giant cells (red arrow), and collection of neutrophils and eosinophils (green arrow).

A 71-year-old male was referred to our outpatient department with rising prostate specific antigen (PSA) levels (6.70 ng/ml, reference range: 0–4 ng/ml). His past medical history was relevant for right lung cancer. His current medical history was relevant for arterial hypertension, which was being treated with zofenopril, diabetes mellitus treated with metformin and psoriatic arthritis under treatment with golimumab plus methotrexate. His family history was negative for PCa and he did not report lower urinary tract symptoms, hematuria or fever. Digital rectal examination of the prostate revealed a focal area of fixed induration involving the right lobe. Urine culture was negative and urinalysis did not reveal abnormal findings. PCa was suspected. A 3-Tesla prostate multiparametric magnetic resonance imaging (MRI) determined the presence of a 4 cm nodular lesion characterized by low signal intensity on T2-weighted sequences and involved both the peripheral and transition zone of the right lobe with extension to the peripheral zone of the mid basal left lobe (Figure 1A). Capsular irregularity, suspicious for extracapsular extension, was also evident. ‘On diffusion-weighted imaging the lesion was characterized by significant signal restriction with low apparent diffusion coefficient (ADC) values (Figure 1B & C).’ Dynamic contrast enhanced images revealed diffuse enhancement post contrast (Figure 1D). A Prostate Imaging Reporting and Data System version 2 score of five was assigned to the lesion, indicating a clinically significant PCa was highly likely to be present. The patient underwent prostate biopsy. Target biopsies revealed aggregates of lymphocytes, plasma cells, histiocytes and epithelioid cells around damaged glands, together with a tubercle-like reaction with multinucleated giant cells, as well as a collection of neutrophils and eosinophils. These aspects were compatible with GP (Figure 2). Non target biopsies demonstrated a marked and extensive chronic prostatitis coexisting with multiple spots of acute prostatitis. The patient underwent observation and PSA spontaneously decreased to 0.70 ng/ml at 6 months follow-up.

## Discussion

GP represents a rare chronic inflammatory condition of the prostate, with autoimmunity being proposed as a potential key pathophysiological alteration involved in the aetiology of most cases [5]. Similarly, psoriasis is an inflammatory disorder characterized by a dysregulation of the innate immune response with autoimmune features [7]. To our knowledge, this is the first published report of GP in a patient with psoriatic arthritis. This case has relevant clinical implications and poses the basis for further investigations. Our findings are consistent with previous studies highlighting the diagnostic challenges due to clinical and radiological overlap between GP and PCa [1,8]. Typically, GP presents with lower urinary tract symptoms, hematuria, fever and chills [4]. Less commonly, it can remain asymptomatic [4]. Interestingly, it is frequently associated with increased PSA levels and finding of areas of prostate indurations at the digital rectal exploration. Rais–Bahrami *et al.* identified features on multiparametric MRI to differentiate GP from PCa in lesions that we previously scored as moderately or highly suspicious for PCa, such as significantly higher ADC values and the absence of high-stage features [9]. However, other authors described low ADC values in patients with GP reaching those found in PCa patients and a high Gleason score, as well as features suggestive of extracapsular extension, mainly in cases of florid GP [3,10]. Therefore, as in the present case, multiparametric MRI findings in patients with GP can strongly mimic PCa. Prostate biopsy and histological evaluation remain the gold standard to differentiate GP from PCa. Histologically, GP is characterized by granulomas, which are clusters of macrophages surrounded by a mononuclear leukocytes and plasma cells [11].

Although very uncommon, the coexistence of GP and PCa has been reported [12]. Therefore, careful follow-up is required after the diagnosis of GP. In most cases, GP resolves spontaneously without treatments [1]. The spontaneous normalization of PSA level observed in the present case further corroborates this evidence. From a speculative point of view, although a direct relationship between psoriatic arthritis, its treatment and GP could not be established based on current evidences, further studies are warranted to evaluate whether the abnormal immunologic signaling involved in psoriatic arthritis plays a direct role in the pathogenesis of GP.

## Conclusion & future perspective

GP is a rare urological entity that should be included in the differential diagnosis in patients with increased PSA levels and findings from digital rectal examination and multiparametric MRI highly suspicious for PCa. The association between psoriatic arthritis and GP we described in the present case underlines the need to further investigate the links between these two autoimmune conditions from both pathophysiological and epidemiological point of views.

**Summary points**

- Granulomatous prostatitis (GP) is a rare chronic inflammatory condition that can mimic prostate cancer at clinical, biochemical and radiological evaluations and characterized by an autoimmune etiopathogenesis in many cases.
- We described a case of GP diagnosed by prostate biopsy in a 71-year-old male with psoriatic arthritis who presented with evidence of rising PSA levels (6.70 ng/ml, reference range: 0–4 ng/ml), focal area of fixed induration involving the right lobe of the prostate at digital rectal examination that appeared at multiparametric resonance imaging as a nodular lesion characterized by a Prostate Imaging Reporting and Data System version 2 score of five.
- Further studies are warranted to evaluate whether the abnormal immunologic signaling involved in psoriatic arthritis plays a direct role in the pathogenesis of GP.
